# Cancer genomics and bioinformatics in Latin American countries: applications, challenges, and perspectives

**DOI:** 10.3389/fonc.2025.1584178

**Published:** 2025-07-09

**Authors:** Erika Sofia Torres-Narvaez, Daniel Felipe Mendivelso-González, Juan Andrés Artunduaga-Alvarado, Oscar Ortega-Recalde

**Affiliations:** ^1^ Departamento de Morfología, Facultad de Medicina e Instituto de Genética, Universidad Nacional de Colombia, Bogotá, D.C, Colombia; ^2^ Department of Pathology, Instituto Nacional de Cancerología, Bogotá, D.C, Colombia; ^3^ Unidad de Biología Computacional y Analítica de datos, Biotecgen S.A.S., Bogotá, Colombia

**Keywords:** cancer genomics, bioinformatics, precision oncology, next-generation sequencing (NGS), Latin America

## Abstract

Next-generation sequencing (NGS) technologies have revolutionized research and precision medicine in patients with cancer. Progress in this area has been accompanied by the development of efficient and robust bioinformatics methods along with computational resources able to handle the growing amount and complexity of sequencing data. Importantly, the implementation of such approaches has not been uniform around the globe and several regions, including Latin American countries, remain lagging behind in cancer genomics and precision oncology. Likewise, numerous studies have highlighted the complexity and particularities of such populations in terms of genetic background, healthcare systems and human and technological resources. In this review, we aim to describe current clinical applications of NGS-based tests, focusing on their bioinformatics analyses and implementation in Latin America. Furthermore, we describe several opportunities for development, perspectives, and challenges that face genomic data analysis in this geographical area. We expect this review to provide an up-to-date overview of cancer genomics and bioinformatics in Latin America, serving as a valuable resource for both local and international cancer researchers.

## 1 Introduction

Cancer is a complex and heterogeneous disease resulting from uncontrolled cell division, leading to abnormal growth and invasion. Currently, a strong body of evidence supports that cancer is predominantly a genetic disease, arising from mutations in genes associated with cell proliferation, survival, migration, and immune regulation (1). These mutations include a wide variety of DNA alterations ranging from point mutations to large genomic rearrangements and are considered critical for tumor development and progress. In addition to being a biologically complex disease, cancer has a devastating global impact in terms of social and economic burden (2, 3). Data from the latest report of the International Agency for Research on Cancer (IARC) showed that in 2022, 20 million new cases and 9.7 million deaths were reported worldwide (4). For Latin America, accounting for approximately 8.4% of the global population, 1.5 million cases and 750,000 deaths were recorded in the same year. Furthermore, model predictions suggest that by 2050 the number of new cases will increase to 35 million worldwide, principally driven by demographic transitions. This global burden has motivated intense research efforts and technological advancements in cancer diagnosis, treatment, and prevention, which in turn have significantly changed the course and prognosis of patients with this disease.

In the last two decades, next-generation sequencing (NGS) technologies, also known as massive parallel sequencing, have revolutionized the field of cancer genomics, providing unprecedented insights into cancer biology and accelerating the development of precision oncology (5). In addition to improving cancer treatment through targeted therapies, these techniques are currently used for molecular diagnosis, disease monitoring, and assessment of predictive biomarkers (6). Furthermore, NGS-based techniques can be employed to detect germline variants, important in hereditary cancer syndromes and pharmacogenomics; somatic variants, useful as tumor biomarkers; and transcriptomic profiles useful in clinical settings. Nowadays, several NGS platforms are available in the market, and significant improvements in cost-efficiency and accessibility have facilitated their adoption in healthcare around the globe (7). While promising, one of the main challenges concerning the implementation of NGS in clinical practice is data analysis (8). NGS studies produce a vast amount of raw data, which must be carefully processed and analyzed to ultimately generate a comprehensive report, useful to the clinical team and patient (8, 9). Data analysis is a non-trivial task and requires highly specialized personnel able to use and develop bioinformatics tools and strategies and correlate the findings with biological and clinical information. In addition, bioinformatics and computational methods are critical for the analysis of such amounts of data and are considered essential for the successful implementation of NGS in precision oncology. Finally, a robust computational infrastructure is required to process high-throughput sequencing data in a timely and effective manner.

**Figure 1:**
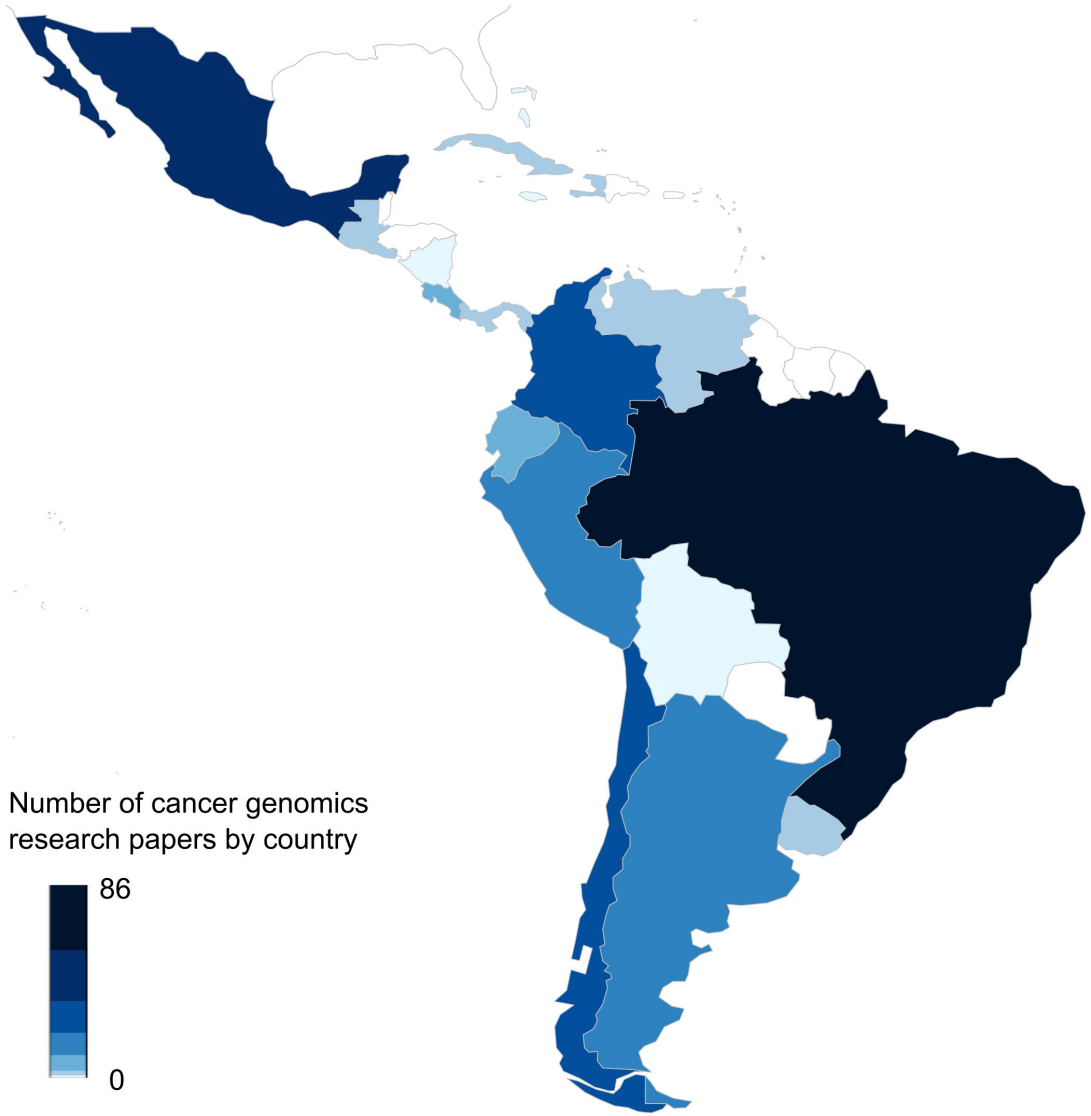
Number of publications in cancer genomics in each Latin American country. The figure shows the results of the frequencies of published papers found in the Scopus database until April 2025 about cancer genomics in each Latin American country. The map was generated using the rworldmap R package (v1.3-8) and the color bar represents the number of articles. The query used for each of the 33 countries was as follows: “TITLE-ABS-KEY(Genomics) OR TITLE-ABS-KEY(Transcriptomics) OR TITLE-ABS-KEY(Epigenomics) OR TITLE-ABS-KEY(Bioinformatics) AND TITLE-ABS-KEY(Neoplasms) OR TITLE-ABS-KEY(Tumor) OR TITLE-ABS-KEY(Neoplasm) OR TITLE-ABS-KEY(Tumors) OR TITLE-ABS-KEY(Neoplasia) OR TITLE-ABS-KEY(Neoplasias) OR TITLE-ABS-KEY(Cancer) OR TITLE-ABS-KEY(Cancers) OR TITLE-ABS-KEY(“Malignant Neoplasm”) OR TITLE-ABS-KEY(Malignancy) OR TITLE-ABS-KEY(Malignancies) OR TITLE-ABS-KEY(“Malignant Neoplasms”)”.

The importance of bioinformatics in clinics is gaining momentum, nevertheless, key challenges remain to be addressed. Among these difficulties, we can highlight the fast-paced development of new methods, applications and technologies, the growing demand for genomic testing, limitations in human and computational resources, and gaps in knowledge amongst healthcare professionals. These challenges are particularly relevant for developing countries with limited healthcare resources, including most Latin American countries. Although not completely integrated into national healthcare systems, several groups and institutions in Latin America are using NGS and bioinformatics tools for clinical oncology. A quick search of cancer genomics studies in each Latin American country in the Scopus database, for example, showed 276 results with Brazil (86), Mexico (57), Colombia (34), Chile (26) and Peru (20) as the main contributors (**Figure 1**). Noteworthy, studies derived from genomic analyses have also stressed differences in genetic background and considerable heterogeneity amongst Latin American populations. Furthermore, these studies have also explored the feasibility, clinical relevance, and limitations that face the implementation of genomic analyses within routine cancer clinical care.

This review aims to present and discuss such clinical applications and, in a broader context, explore the challenges and opportunities of cancer genomics bioinformatics in the region. In the first part of this review, we will describe and illustrate examples of clinical applications of bioinformatics methodologies to study cancer genomics with special emphasis on the Latin American region. Next, we will focus on current challenges that hinder the successful implementation of bioinformatics platforms and propose possible solutions to address them. Finally, considering the vertiginous development of new technologies and bioinformatics approaches, we will present active areas of research that we consider will have a significant clinical impact in the near future.

## 2 Current clinical applications

NGS and bioinformatics tools are increasingly being used in the evaluation of cancer patients, bridging the gap between molecular data and oncology decision-making. Despite its relatively recent emergence, these tools have become increasingly available and utilized in clinical settings, particularly germline and somatic mutation testing and the analysis of transcriptomic profiles. These applications are reshaping cancer care worldwide, including the Latin American region, fostering precision medicine tailored to diverse populations.

### 2.1 Germline cancer testing

Germline cancer testing plays a critical role in identifying hereditary cancer syndromes, facilitating personalized preventive strategies such as enhanced surveillance, lifestyle modifications and prophylactic interventions (10). In addition, this strategy has important implications for patient screening, diagnosis, prognosis and treatment, which can be extended to other family members or communities. Currently, several guidelines and consensus include germline testing recommendations for specific tumors and high-risk patients (11, 12). Multiple recent studies have even explored the utility of universal germline cancer testing, this is cancer genetic testing for all cancer patients, providing strong evidence of its usefulness in clinical oncology and medical genetics (13–15). A pan-cancer study performed by Stadler et al., for example, analyzed 11,947 patients with advanced cancer, finding that 17% harbored likely pathogenic or pathogenic germline variants and 9% had a germline variant with therapeutic implications (13). Another study prospectively analyzed a cohort of 2,984 patients finding pathogenic variants in 13.3% of the cases, including 9.4% located in moderate- and high-penetrance cancer susceptibility genes (14). Furthermore, this study found that 28.3% of the patients with high-penetrance variants had modifications in their treatment based on their findings. These and other studies highlight the usefulness of germline cancer testing in patients with cancer and provide a strong foundation for the application of this approach in clinical practice.

**Figure 2:**
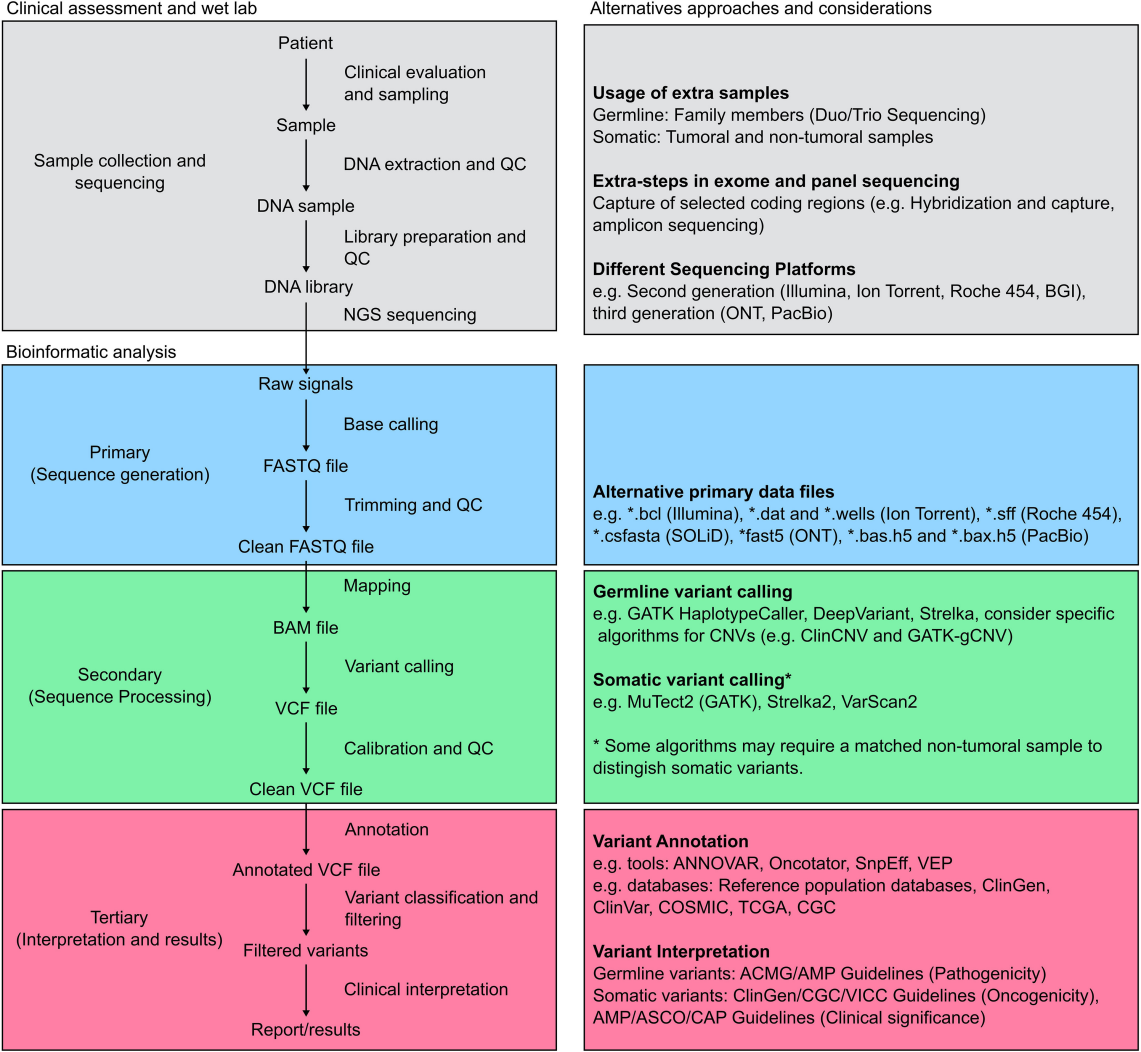
Standard pipeline for NGS analysis. The figure depicts a general pipeline for the analysis of NGS data along with complementary information. The grey boxes outline the process of clinical data and sample collection. Additional samples may be collected from family members and matched samples, and multiple sequencing approaches and platforms are currently available. The bioinformatic pipeline is then divided into three major steps: First, sequence generation (blue boxes), where raw sequencing data obtained from the equipment is converted into a sequence file format, most commonly FASTQ. Following trimming and quality control (QC), clean FASTQ files are mapped to a reference sequence and stored during the sequence processing step (green boxes). Different algorithms, optimized for germline or somatic variants, can then perform variant calling. Finally, variant annotation and interpretation (red boxes) are performed using a semi-automatic approach aiming to generate a clinical report or meaningful results according to the study goal.

Currently, several NGS-based strategies to identify germline variants associated with cancer are available, including targeted sequencing, exome sequencing and whole genome sequencing. These approaches vary in cost, diagnostic yield and analysis complexity, nevertheless, targeted sequencing, also known as gene-panel sequencing, is the most commonly used method to identify cancer-related variants in clinical settings (16). Any of these approaches involves a series of sequential steps that begin with a detailed clinical evaluation prior to ordering the test (17). This initial evaluation is not only critical to indicate opportunely the test but also for genetic counselling and interpretation purposes. In most cases, DNA is extracted from blood or saliva and sequencing library preparation is performed using standardized protocols, specific to the NGS platform to be employed (18). Regardless of the NGS technology used, sequencing raw data is primarily stored in FASTQ format and follows a standardized bioinformatics pipeline illustrated in **Figure 2**. First, the raw FASTQ files are trimmed to remove adaptors and low-quality bases and reads. Next, clean FASTQ files are mapped to a reference genome. The aligned reads are stored in a format known as SAM (Sequence Alignment/Map) which is commonly compressed in the binary format BAM (Binary Alignment Map). Next, variant calling is performed using a variant caller like GATK or DeepVariant incorporating bioinformatics best practices such as deduplication and recalibration (19, 20). Additionally, several bioinformatics tools allow the detection of copy number variants (CNV) using NGS data (21, 22). The variants obtained are stored in a format called VCF (Variant Call Format) and annotated. The next step involves a semi-automatic filtering of the variants identified. This process includes excluding variants based on allele frequencies, as those that are common in the general population are less likely to be associated with hereditary cancer syndromes, and using different sources of data such as bioinformatics predictions, functional analyses, genetic databases and family segregation information to prioritize and classify variants. Given the heterogeneity of data sources and variant interpretations, the American College of Medical Genetics and Genomics (ACMG) and the Association for Molecular Pathology (AMP) issued a guideline for classifying genetic variants using a five-point scale to assign pathogenicity in 2015 (23, 24). The scale ranges from benign (not disease causing) to pathogenic (disease causing), with intervening scores of likely benign, variant of uncertain significance (VUS) and likely pathogenic. Although several updates and alternatives have been proposed and implemented, the ACMG guidelines remain the most widely used classification system (25, 26). Nowadays, several bioinformatics companies offer automatic software and platforms to facilitate this process, nevertheless, it is important to highlight that given the clinical implications of these tests, all the steps and the generation of the final report should be supervised by a multidisciplinary team of physicians, geneticists, molecular biologists and bioinformaticians (27). In addition, similar to wet-lab protocols, oncology clinical practice guidelines firmly advocate for the validation of bioinformatics pipelines in local settings (28).

The adoption of NGS platforms for germline cancer genetic testing in the Latin American region has been increasing over the last years. Interestingly, several studies have shown considerable differences between and within populations in this region. A recent study, for instance, analyzed 24,075 Latin American individuals undergoing testing for hereditary breast and ovarian cancer, finding that between 9.1% - 18.7% harbored pathogenic variants (29). This study included patients from Mexico, Central America, the Caribbean, South America, and US Hispanics reporting also a higher diagnostic yield in patients living in the Latin American region compared to US Hispanics. In another study, 403 individuals meeting the criteria for Hereditary Breast and Ovarian Cancer syndrome from Argentina, Colombia, Guatemala, Mexico, and Peru were analyzed for germline variants (30). The prevalence of pathogenic variants across these countries underscored the genetic heterogeneity of Latin American populations, with Argentina showing the highest prevalence at 25% and Colombia the lowest at 13%. Several examples of other studies in Latin American countries and their main results are presented in **Table 1** (31–40). These results may stem from the complex genetic admixture in the region but also from differences in lifestyles and environmental factors, public health policies, and technical aspects (41). Regarding laboratory and bioinformatics practices in the Latin American region, these studies show significant variability. Some centers limit testing to specific genes, such as *BRCA1* and *BRCA2* for breast and ovarian cancer, while others employ NGS panels that include around 25 to >200 genes (34, 38). Remarkably, the guidelines and parameters used to define the clinical significance of genetic variants are not completely standardized across studies, potentially leading to inconsistencies in variant classification and difficulties in the comparison of results between centers and countries, creating challenges in establishing conclusions (42). On the other hand, bioinformatics methods and tools are not always presented in the studies and clinical reports, limiting their reproducibility and comparability. It is important to highlight that bioinformatics best practices and guidelines in clinical settings are increasingly relevant as these tests are more widely adopted worldwide (28). Finally, the genetic and bioinformatics “literacy” amongst clinical practitioners to interpret and use the results, a topic that will be discussed in more detail below, is a challenge probably underestimated in our region that significantly affects the utility of these tests (43).

**Table 1:** Examples of germline NGS studies in patients with cancer from the Latin American region.

Type of cancer	Country and Year	Methods	Bioinformatics analysis	Main results	Reference
Breast and ovarian	Argentina, 2016	940 patients with HBOC were analyzed by MLPA and NGS sequencing using a customized panel for *BRCA1* and *BRCA2* variants.	Variant analysis used the Breast Cancer Information Core Internet Website (BIC) and/or predictive algorithms: Align-GVGD*, SIFT*	179 deleterious variants (19%) including 5 rearrangements and 22 novel variants were found. Overall, only 2.87% mutations were recurrent, suggesting a limited usefulness of tests assessing punctual variants.	(31)
Breast	Brazil, 2018	157 individuals at risk for hereditary cancer were analyzed using three different NGS panels (33 genes, 94 genes/284 SNVs and 11 genes)	Pathogenic and VUS variants identified went through quality analysis, database query and prediction of pathogenicity using PolyPhen-2*, SIFT/PROVEAN* and MutationTaster*.	19 variants found in 17% of individuals, 15 P and 4 VUS. 68% of the mutations were found in *BRCA* genes and 32% in moderate-risk genes.	(32)
Breast	Brazil, 2018	7 patients with HBC negative for variants on major risk genes (*BRCA1/2*, *TP53*, *CHEK2* c.1100delC) were analyzed using WES.	Variants were called using GATK*, annotated with VarSeq* and then filtered by quality and population frequencies. Functional prioritization and prediction algorithms were used to identify novel variants potentially associated with HBC.	Two causative variants were found in *ATM* and *BARD1* genes along with 4 VUS in previously known HBC genes. This study proposed 12 candidate genes for Brazilian population highlighting *NOTCH2, ERBB2, MST1R, RAF1, ERCC1* and *SLX4*.	(33)
Breast	Colombia, 2018	85 patients with criteria for HBOC were analyzed by an NGS commercial panel including 25 genes.	Not reported.	19 patients (22.4%) carried deleterious germline variants, with *BRCA1/2* variants accounting for 17.6%. A low frequency (1.2%) of known Colombian founder variants was identified.	(34)
Breast and ovarian	Mexico, 2018	Analysis of two groups of high-risk (27) and cancer (300) patients using an NGS panel including 143 genes.	Secondary and tertiary analysis using BWA*, GATK*, ANNOVAR* and InterVar*. Variant classification manually curated according to ACMG guidelines.	Pathogenic variants in 23 genes with a higher contribution of other susceptibility cancer genes (54%) than *BRCA1/2* (46%). High frequency of Mexican founder mutations (e.g. del exons 9-12, p.G228fs in *BRCA1*).	(35)
Endometrial	Brazil, 2020	Analysis of *MLH1*, *MSH2*, *MSH6*, *PMS2*, and *EPCAM* genes by NGS on 37 patients with endometrial carcinomas.	Bioinformatics analyses using GATK*, ANNOVAR* and VisCap*. Classification based on ACMG guidelines using VarSome search Engine.	10 samples were positive for P/LP variants. 40% were detected on *MSH6* gene and 8 were novel variants. Seven variants were classified as VUS.	(36)
Hereditary cancer risk syndromes	Brazil, 2022	Genotyping of 1682 individuals with multiple ethnicities using NGS panels including between 37 and 143 genes.	Variant calling and analysis included GATK*, annotation with ANNOVAR* and in-house databases. Variant classification according to ACMG guidelines.	305 individuals (18.1%) carried at least one P/LP variant across 32 genes. Most variants were found in *BRCA1/2, MUTYH, PALB2, TP53* and MMR genes. Additionally, 753 (44.8%) had at least one VUS variant.	(37)
Mostly colorectal and endometrial in suspected LS	México, 2022	NGS panels of 263/322 genes used in 412 patients with suspected LS.	Bioinformatics methods not reported. Variant classification according to ACMG guidelines.	27.1% variants were found on a gene of the MMR pathway while 30.4% were present on *CHEK2, APC, MUTYH, BRCA1*, and *BRCA2* genes.	(38)
Hereditary Cancer Syndromes	Mexico, 2023	NGS panel including 30 or 84 genes in 205 individuals with suspicion of HCS or relatives.	Bioinformatics analysis performed with Sequencing Analysis* and SeqScape*. Variant classification according to ACMG guidelines.	Among the probands, 85 (63.5%) had at least one P/LP germline variant. Most variants were found in *BRCA1* and *MLH1*.	(39)
Breast	Colombia, 2024	WES and MLPA on 400 unselected women with breast cancer.	Variant calling and analysis included GATK* and annotation with VarSeq*. Variant classification according to ACMG guidelines.	24 (6%) patients had P/LP variants. Most variants were found in *BRCA2* (2.5%)*, ATM* (1.25%) and *BRCA1* (0.75%). 1.75% of recurrent variants were found in *BRCA2* and *ATM* genes.	(40)

ACMG, American College of Medical Genetics and Genomics; HBC, Hereditary Breast Cancer; HBOC, Hereditary Breast and Ovarian Cancer; dMMR, DNA mismatch repair deficiency; LP, Likely Pathogenic; LS, Lynch Syndrome; MLPA, Multiplex ligation-dependent probe amplification; MMR, Mismatch Repair; P, Pathogenic; VUS, Variant of Uncertain Significance; WES, Whole Exome Sequencing; *Bioinformatics algorithms/software.

Despite the considerable advances in the implementation of hereditary cancer programs and genetic testing in Latin America, these have been heterogeneously implemented in different countries. This heterogeneity may be primarily due to structural differences in healthcare systems and limitations in human and economic resources (44). In Chile, for example, despite national recommendations advocating for universal genetic testing for patients with breast cancer, the country is far from achieving this objective. A study conducted by Acevedo et al. in two centers in Santiago, Chile during 2023 revealed that only 15% of patients with breast cancer meeting the criteria for genetic testing underwent this procedure (45). Furthermore, this study highlighted the disparities in access between private and public institutions. In Mexico, the public health system does not cover the costs of germline cancer testing and some studies mentioned the dependency on research projects to perform genetic testing (46). This approach possesses many challenges for the sustainability of germline cancer screening programs. In Colombia, germline testing is covered by the health insurer as it is considered a diagnostic procedure (47). In this country, a recent study by Sierra-Díaz et al., found that 6% of the Colombian women with unselected breast cancer had germline mutations in high-penetrance cancer susceptibility genes (40). Interestingly, the numerous challenges experienced in these countries have also generated opportunities to optimize resources and improve healthcare systems. For instance, one center in Mexico has successfully implemented a germline cancer testing service that includes telemedicine (46). This innovation enabled patients from rural and underserved areas to access genetic counseling and testing, effectively bridging a critical gap in genomic medicine. Similarly, the establishment of national cancer programs in countries such as Chile and Colombia has facilitated the gradual integration of germline screening programs in healthcare systems (48, 49). Notably, the development of robust and accessible cancer genomics programs has progressed more slowly than anticipated, highlighting the need for sustained efforts to overcome the existing barriers.

### 2.2 Somatic cancer testing

The identification of driver mutations in cancer genomes is considered one of the pillars of precision oncology (5). These mutations play a critical role in cancer development and are valuable biomarkers for diagnosis, prognosis and targeted therapy. In contrast to low-throughput molecular tests, NGS-based somatic cancer approaches can simultaneously analyze multiple gene regions and even the entire genome. Currently, leading organizations such as the European Society for Medical Oncology (ESMO), have included evidence-based recommendations for the use of tumor NGS in patients with advanced non-squamous non-small-cell lung cancer (NSCLC), prostate cancers, breast cancers, ovarian cancers, among other tumor types (50). These recommendations are based on multiple lines of evidence, for instance, a recent comprehensive review on the clinical impact of NGS tests for the management of advanced tumors showed that progression-free survival and overall survival among patients who received NGS-guided cancer treatment were significantly longer across multiple tumor types (51). Another large study assessing 109,695 patients with solid tumors found that among the most common cancer types, predictive, prognostic, and diagnostic markers were reported in 51.2% of tumor profiles, and 89.2% had genomic results that could inform guided therapies (52). While the decision to choose between different NGS test options may be challenging and relies on multiple factors including tumor biology characteristics, test availability, and cost-effectiveness, somatic cancer studies are critical to improving cancer care (53). Furthermore, numerous clinical trials matching specific genomic profiles to novel cancer therapies have shown that the growing knowledge gained through cancer research is continuously being integrated at this level, providing a powerful tool in translational medicine. Despite its relevance, one of the main bottlenecks of these approaches is the analysis and interpretation of the large amount of data generated through NGS, constituting a potential barrier to wide clinical adoption (54).

Several parallels can be drawn between germline and somatic cancer data analysis, nevertheless, the identification of somatic variants faces challenges. Regarding the tumor sample, DNA can be obtained from fresh samples, liquid biopsies, or formalin-fixed paraffin-embedded tissues (FFPE). Importantly, these samples may contain different amounts of genetic material and proportions of normal and tumor cells, described as the purity of the sample, which affects further analysis (6). Moreover, clonal evolution in cancer cells may result in genetic intratumoral heterogeneity, which is not always well represented in the sample taken and could lead to false negative results. In light of these confounding factors, it is suggested that clinical reports include sample quality parameters along with sequencing quality information (19).

Another important consideration is the correct distinction between germline and somatic variants. This is usually achieved through the direct comparison of tumor samples and patient-matched normal tissue samples, such as peripheral blood (19). When the study relies only on tumor samples, the origin can be inferred using variant allele frequencies (VAF), databases of recurrent germline variants and specific algorithms. While tumor-only studies are more cost-effective than matched tumor-normal sequencing, it carries inherent limitations. This is due to the potential misclassification of germline variants in population databases, variability in tumor purity, and differences in intratumor heterogeneity across specimens (55). Finally, to enhance sensitivity for detecting somatic variants, it is necessary to choose a specific sequencing depth, defined as the average number of aligned reads at a given genomic position. This will depend on the threshold defined to the limit of detection (LOD), tolerance for false positive/false negative results, and sequencing error rates (56). For example, increasing sequencing depth beyond standards used in germline studies is recommended for low-frequency somatic variants (19, 57). Although currently there is no consensus on the optimal sequencing depth in the context of somatic variants, some targeted somatic panels have recommended ranges between >500X for LOD of 5% to > 1000x for low tumor cellularity samples (56).

The bioinformatics analysis of NGS-based somatic cancer techniques follows similar steps to NGS germline techniques. A global overview of this pipeline is presented in **Figure 2**. Importantly, somatic variant calling remains a challenging task due to the cancer genome complexity and several bioinformatics tools have been specifically designed to optimize the identification of somatic variants, including MuTect2, Strelka2 and VarScan2 (58–60). These methods can integrate somatic and germline information to tackle biological and technical issues such as low VAF and low sample purity. Intriguingly, several studies comparing these tools have shown differences in performance, suggesting that the combination of techniques could maximize somatic variant discovery (61). Alternatively, when only tumor sequencing information is available, general or specific variant callers must be optimized to detect somatic variants, taking into account the potential issues previously mentioned (19, 62). The variants obtained from this step are then stored in VCF format for conducting tertiary analysis, including clinical interpretation and correlation. In addition to general and germline databases, numerous somatic and cancer-specific resources can be used to classify and interpret the findings, and somatic cancer reports often include more information about specific variants (63, 64).

Two concepts are particularly relevant in somatic cancer analyses and reports: oncogenicity and clinical significance. Oncogenicity, defined as “the pathogenicity of the variant in the context of a neoplastic disease”, is classified according to a joint consensus of the Clinical Genome Resource (ClinGen), the Cancer Genomics Consortium (CGC), and the Variant Interpretation for Cancer Consortium (VICC) (65). This system classifies the variants into 5 categories: oncogenic, likely oncogenic, variant of uncertain significance (VUS), likely benign, and benign, based on an evidence point system including population, functional and predictive data, cancer hotspots, and computational evidence. The evidence strength in each data type adds or subtracts points to the final score of each variant allowing its categorization. Second, clinical significance, defined as the variant’s impact on clinical care in terms of diagnosis, prognosis, and/or therapeutic biomarkers (55). Clinical significance is classified according to an evidence-based system proposed by a joint consensus of the AMP, the American Society of Clinical Oncology (ASCO), and the College of American Pathologists (CAP). This system uses different sources of information, including guidelines, FDA approvals, dedicated databases, and computational predictions, to classify variants in 4 Tiers: Tier I, variants with strong clinical significance; Tier II, variants with potential clinical significance; Tier III, variants with unknown clinical significance; and Tier IV, variants that are benign or likely benign. Given the growing amount of information and continuous updates related to clinical associations, knowledge databases are created to integrate the data. Examples of these efforts include the Cancer Genome Interpreter Cancer Biomarkers Database (CGI), Clinical Interpretation of Variants in Cancer (CIViC), Jackson Laboratory Clinical Knowledgebase (JAX-CKB), OncoKB and the Precision Medicine Knowledgebase (PMKB), among others (66–70). Harmonization of this data to obtain reproducible results using different tools is necessary to integrate and standardize the information included in the final clinical report (64).

The implementation of somatic NGS-based analyses in cancer research and clinical practice in Latin America has been slow but steady. By 2017, it was estimated that more than 221 NGS platforms were available in the region and 272 articles were reported to have Latin American authorship associations, with Brazil and Mexico as major contributors (71). Furthermore, the incorporation of NGS-based technologies has enabled the transition from specific mutation methods to comprehensive cancer genomics studies, allowing to detect novel clinical associations in these populations. A recent study performed by the CLICaP consortium, for example, analyzed the genomic landscape of primary resistance to Osimertinib among Hispanic patients with EGFR-mutant non-small cell lung cancer, showing that specific findings such as commutations, and the presence of the mutations EGFR p.T790M and p.L858R are associated with therapeutic responses and patient outcomes (72). Another recent study in Chile, suggested potential differences in driver mutations for Chilean patients with colon cancer when compared to cohorts with different ancestries (73). Given their epidemiological relevance, prostate (15%), breast (14%), colorectal (9%), lung (7%) and gastric (5%) cancers are amongst the principal focus of research and precision oncology initiatives in this region (74). Some examples of these studies and their conclusions are presented in **Table 2** (75–83). These findings are particularly relevant in clinical oncology due to the underrepresentation of Latin American populations in precision medicine studies and databases. Interestingly, some studies have also focused on the design and validation of cost-effective NGS platforms optimized for our region (84). In addition to optimizing resources, these types of studies are remarkable in terms of technological appropriation and open source bioinformatics solutions.

**Table 2:** Examples of somatic NGS studies in patients with cancer from the Latin American region.

Type of cancer	Country and year	Methods	Bioinformatics analysis	Main results	Reference
Cervical	Guatemala, Venezuela, and Mexico, 2016	Genomic characterization of the disease with exome (100X) and ultra-deep targeted sequencing (500X) in 24 tumor samples	A custom analysis workflow was performed including variant calling, CNA identification and annotation for exome and Targeted Sequencing with GATK*, TSVC* among others.	Higher frequency of mutations in the PI3/AKT pathway with different distribution compared to other cancer types. Mutational cluster observed in the helical domain (E542, E545) of *PIK3CA* gene, relevant for therapy.	(75)
Breast	Chile, Colombia, Costa Rica, and México, 2019	Characterization of the molecular landscape in breast cancer from 126 premenopausal women using targeted deep sequencing (1000X) and exome for paired samples	Somatic variant calling was performed with ITVC* and Strelka*, Annotation with Annovar*, sequencing artifacts were detected with MutSpec*. Pathway analysis was derived from ConsensusPathDB** and cancer gene identification with COSMIC** and IntOGen**.	*PIK3CA* (32.5%) and *TP53* (21.4%) were the most mutated genes along with *AKT* (9.5%), with concordant classic hotspot. Difference in the expected distribution of TP53 substitution G:C>T:A 1.5 vs. 3.3. Mutational patterns shows alterations on signal transduction pathways and signatures related to DNA repair pathways.	(76)
Lung	Brazil, 2020	Use of 513 comprehensive genomic profiling results to describe somatic and co-occurring mutations of NSCLC patients with tumor (457) and ctDNA (56) samples.	Unique samples for each tumor stored on Foundation Medicine database including genomic data and tumor mutational burden (TMB). No bioinformatics analysis reported	Most common mutations were identified in genes *TP53, KRAS, EGFR, STK11, PIK3CA, ALK, BRAF, ERBB2* concordant with previous prevalences for driver mutations. Co-mutations were found for *TP53* (e.g. association of *TP53* p.R337H with *EGFR* and *ERBB2* mutations).	(77)
Pancreas	Mexico, 2020	Exome (50-100X) and transcriptome paired sequencing to characterize PDAC in 4 Mexican patients.	An in-house workflow was created using the following algorithms: MuTecT* (SNVs), IndeLocator* (Indels). Annotation with Oncotator*, CAN analysis with ControlFreec* Filters: Oncogenic driver Genes list**	Mutations identified in three previously associated genes *HERC2, CNTNAP2* and *HMCN1*. Of note, there is an absence of mutations in *KRAS* which are common among Caucasian populations.	(78)
Lung, unknown primary, female reproductive system, among others	Colombia, 2023	NGS genomic comprehensive profiling of different types of 125 solid tumors to identify actionable mutations.	Not reported	Actionable mutations identified in 58 cases (46.4%). 22.1% of genomic alterations were classified as Tier I, 11% as Tier II and 7.3% as Tier III.	(79)
Central nervous system	Brazil, 2023	Whole-exome sequencing (100X) was performed on paired samples of an atypical choroid plexus papilloma and a choroid plexus carcinoma.	Bioinformatics analysis was performed using Mutect2*, PINDEL* and NEXUS* for variant identification. Annotation of genes include ANNOVAR* and filtering using population frequencies.	Two variants of clinical significance were found in *BIRD1* and *TP53*. The high VAF >90% in a *BIRD1* variant was associated with and additional *CNA* loss.	(80)
Prostate	Colombia, Mexico, Peru, Argentina, Chile, 2024	Genomic characterization of metastatic prostate cancer on 348 patients using a Multigene Panel.	Commercial pipeline SOPHiA DDM*. ESCAT* platform was implemented to determine actionability.	In 16 patients (15.8%) an actionable somatic mutation was identified with no difference among hormone sensitive and castration-resistance prostate cancer.	(81)
Lymphoma	Mexico, 2024	Description of the genomic landscape in 185 patients using a customized NGS panel including 79 genes to perform clinical and outcome correlations	Variant identification analysis used TSVC*. The process of Annotation and Interpretation was performed with population databases and predictive algorithms. CGI** was used for the identification of driver mutations.	110 patients (59.5%) had one or more driver mutations. The genes *TP53, EZH2, CREBBP, NOTCH1*, and *KMT2D* genes were the most common mutated. No correlation with survival was found.	(82)
Gastrointestinal, lung, central nervous system, sarcoma, among others.	Colombia, 2024	Characterization of somatic profile and its effect on treatment selection with the TruSight Oncology 500 panel in 103 samples.	Commercial pipeline SOPHiA DDM* software was reported for bioinformatics analysis,	Most frequently somatic mutated genes were *TP53*, *KMT2C*, and *NCOA3*. *ATR* c.2320dup (p.Ile774fs) was the most common variant found among samples and colon cancer showed the highest mutation frequency.	(83)

av, Average; CAN, Copy Number Alterations; ctDNA, circulating tumor DNA; NSCLC, non-small cell lung cancer; paired samples, including blood and tumoral sample; PDAC, Pancreatic ductal adenocarcinoma; SNVs, single nucleotide variants; VAF, Variant allele frequency; X, depth of sequencing; *Bioinformatics algorithms/software, **Genomic databases

Overall, diverse studies focused on somatic cancer studies in Latin America show high heterogeneity in technical and analytical aspects. Regarding mutation detection strategies, although multiple techniques are currently available, the transition to NGS-based technologies is accelerating (85). This transition is associated with increasing testing costs, nevertheless, several studies examining the cost-effectiveness of this approach suggest that robust analyses should be conducted in specific scenarios and that NGS-based tests are cost-effective in multiple clinical settings (86, 87). FFPE samples are the most common tissue analyzed due to their ease of storage and cost-efficiency, even though they are prone to DNA damage associated with the technique, time of storage and quality of the material and protocols (88). In this regard, it should be highlighted that high-quality materials and methods should be prioritized to optimize DNA recovery. Different NGS sequencing technologies, strategies and bioinformatics pipelines have been used in these analyses, including commercial and in-house gene panels and bioinformatics workflows. Despite the importance of quality parameters, these are not always included in clinical reports and studies. Similarly, there is still a large heterogeneity in the implementation of the oncogenicity and clinical significance parameters. Finally, the impact of these tests on clinical decisions has been rarely explored in our region. Given their importance in clinical practice, it is expected that somatic cancer studies will become standard-of-care in oncology and will dramatically improve the outcome of patients with cancer (50, 89).

### 2.3 Transcriptomic profiles

The transcriptome is the entire set of expressed RNA in a particular cell or population of cells at a specific time point. In contrast to the genome, which is considerably more stable in time, the transcriptome is highly dynamic, responding to environmental stimuli and endogenous cues (90). In cancer, gene expression studies have been critical to understanding tumor biology and in clinical practice (91, 92). Historically, these methods include Northern blotting and reverse transcriptase quantitative polymerase chain reaction (RT-qPCR). RT-qPCR, for example, can be used to detect specific gene fusions and quantify the expression of a limited number of genes (93). Although highly sensitive and specific, these techniques only allow the assessment of a determined and reduced number of transcripts or alterations. Later, the introduction of expression microarray enabled the analysis of a considerably larger number of genes, expanding the potential use of transcriptomic data in clinics (94). Currently, several commercial platforms, for example, Oncotype DX™ (Genomic Health), MammaPrint™ (Agendia) and EndoPredict™ (Myriad Genetics) offer gene expression-based analyses for clinical purposes (95–97). Despite their importance, the clinical usage of transcriptomic techniques remains limited due to several factors, including performance in different clinical settings complexity of the analyses, uncertain clinical interpretation and cost-effectiveness (98–100).

As a result of multiple technological and computational advances, NGS of RNA (RNA-seq) has been consolidated as a robust and versatile method for the analysis of tumor transcriptomes. In contrast to DNA sequencing, RNA-seq is primarily a qualitative and quantitative method (101). On the one hand, it allows the detection of isoforms, variants, aberrant splicing and gene fusions. On the other hand, it can be used to accurately measure gene expression levels, resulting in a robust and unbiased approach to studying the transcriptome and indirectly, the genome. Several types of RNA-seq are currently available, nevertheless, whole transcriptome RNA-seq (WTS) and targeted RNA-seq are the methods more commonly used for clinical purposes (102). WTS is a nonselective technique optimal for the discovery of new biomarkers and obtaining a complete picture of the transcriptome and being used for the detection of novel gene fusions, assessment of VUS and molecular characterization of transcriptomic profiles. Although versatile, the main setbacks of this technique are the quality requirements of the sample to be assessed, the sequencing depth to detect lower abundance events, and the costs and complexity of data analysis. Some of these limitations can be fixed by limiting the number of transcripts to be assessed through targeted RNA-seq. This method involves the selection and sequencing of specific transcripts of interest, reducing costs, making analyses more simple, and increasing the sequencing depth of informative events. As expected, the main setback of this approach is the inability to assess genes or events outside the targeted panel. In addition, multiomic approaches, integrating, for example, genomic and transcriptomic sequencing, have emerged as powerful tools to understand tumor complexity and ultimately improve cancer care (103).

The process of RNA-seq begins with the isolation of RNA from the tumor sample and library preparation. These steps, along with sample collection, are critical for obtaining high-quality data and have been extensively discussed in previous reviews (104, 105). An important point about RNA-seq data analysis is that there is not an optimal bioinformatics pipeline for all applications and scenarios in which this method can be used, therefore, these steps should be optimized accordingly (106). Overall, three major phases can be distinguished. First, a pre-analytical phase, which includes an adequate experimental and sequencing design. Once sequencing is performed, this phase includes raw reads quality control and other steps to ensure that data quality is appropriate, for example, read and alignment quality or assessment of batch effects. The second phase, or core analysis, begins with mapping reads to a reference genome, transcriptome or alternative database. This step may include quantitation of transcripts, transcript discovery and differential expression analysis. Finally, an advanced analysis phase can be performed according to the study goals. This phase may include data visualization, gene-fusion discovery, data interpretation, and integration with other techniques and data, including DNA sequencing and clinical information. It should be highlighted that in comparison to DNA-seq, RNA-seq data analysis is less standardized and might be more challenging, particularly in clinical scenarios.

Recently, numerous studies have shown the utility of RNA-seq in clinical settings, including pediatric low-grade glioma, acute lymphoblastic leukemia and breast cancer (92, 107–109). Hardin et al., for example, conducted a multicenter study analyzing 125 samples of patients with low-grade glioma using RNA-seq (107). Interestingly, the authors found that in addition to detecting genomic alterations previously found by other techniques, RNA-seq identified driver mutations not previously detected in 27 cases, 81% of them classified as actionable. Another study by Pleasance et al. combined whole genome and transcriptome sequencing analysis (WGTA) to study 570 patients with advanced or metastatic cancer of diverse etiologies (92). In this study, the authors identified clinically actionable targets for 83% of patients, of whom 37% received WGTA-informed treatments, and 46% of them resulted in clinical benefit. Remarkably, RNA-seq data was highly informative, being useful in 67% of WGTA-informed treatments. These studies highlight the transformative potential of integrating this technique into current cancer diagnostic methods to enhance patient outcomes.

In Latin America, the implementation of RNA-seq and expression arrays for analyzing solid tumors is gaining relevance, particularly in academic settings and specialized reference centers. These techniques have facilitated the identification of specific biomarkers and the molecular characterization of prevalent cancers in the region, including breast, lung and gastric cancers. Given its clinical relevance, multiple studies have focused on understanding the biological landscape of breast tumors in Latin American women (110–112). Romero-Cordoba et al., for instance, provided a detailed genomic and transcriptomic characterization of 204 breast tumors in Hispanic and Mexican women, contrasting their genomic context with patients from African, African American, Asian, and European ancestries and revealing unique molecular features in local populations (111). Another study conducted by Llera et al. used expression arrays to analyze a multi-country cohort of 1071 breast cancer patients as part of the Molecular Profile of Breast Cancer Study (MPBCS), identifying intrinsic subtypes using thePAM50 classification system and revealing similarities and differences in this cohort when compared to other studies (110). Other examples of studies in this and other cancer types are presented in **Table 3** (110–116). In addition to solid tumors, RNA-seq has gained importance in the study of hematological malignancies, including non-Hodgkin lymphomas (NHL) and acute lymphoblastic leukemia (ALL). The Epidemiology of Lymphomas in Latin America (ELLA) cohort study, led by the “Grupo de Estudio Latinoamericano de Linfoproliferativos” (GELL), exemplifies these efforts by investigating the genomic and immunologic landscapes of NHL subtypes and developing prognostic models tailored to regional populations (117). Similarly, a retrospective multicenter cohort study by the Mexican Inter-Institutional Group for the Identification of the Causes of Childhood Leukemia (MIGICCL) utilized RNA-seq to analyze 49 children with ALL (114). This study identified a high prevalence of recurrent and novel gene fusions, including *DUX4* and *CRLF2* alterations, which correlated with poor outcomes, highlighting disparities in survival compared to global cohorts. Together, these initiatives demonstrate how transcriptomic analysis is unveiling critical molecular insights, advancing precision oncology, and addressing unique challenges in both solid and hematological malignancies across Latin America.

**Table 3:** Examples of transcriptomic studies in patients with cancer from the Latin American region.

Type of cancer	Country and year	Methods	Bioinformatics analysis	Main results	Reference
Lung cancer	México, 2016	Gene expression microarrays analyzing primary lung adenocarcinoma in 27 cancer patients with wood smoke or tobacco exposure.	Statistical analysis for differential expression: R and Bioconductor. oligo, sva, limma package*. Biological networks and functional analysis: QIAGEN’s Ingenuity Pathway*.	Gene expression profiling identified 57 DEG.	(113)
Breast cancer	Mexico, 2021	WES and genome-wide microarrays (Affymetrix) for molecular profiling and DNA copy-number analysis in 204 patients.	Genomic analysis: MutSigCV*, GISTIC* and RMA*. Transcriptomic analysis: PAM50*. Immunologic evaluation: ssGSEA Multiomics analysis: MEMo	Patients had more Luminal A tumors (43%) and fewer basal tumors. Higher tumor mutational burden than other groups. 78% had driver mutations, with AKT1 E17K showing a high prevalence (8%).	(111)
Breast cancer	Argentina, México, Brazil, Uruguay and Chile, 2022	Two-color microarrays and immunohistochemistry analyzing 1071 patients with locally advanced breast cancer.	Microarray analysis: Agilent gene expression platform* and HsAgilentDesign026652.db*. Normalization and Batch Correction: Agi4x44.2c* sva*. Pathway enrichment analysis: GSEA*, GSVA* and MetaCore*. DEG: limma* Transcription factor analysis: DoRothEA* and VIPER*.	Luminal A tumors had the best prognosis and basal tumors the worst, with PAM50 stratifying risk and transcriptomics showing higher proliferation in Luminal B, HER2E, and basal.	(110)
Acute lymphoblastic Leukemia	México, 2022	RNA-seq in 49 patients	Fusion detection: FusionCatcher* Differential expression: DESeq2*.	65.3% of patients had at least one fusion, with 31 detected in total. 14 recurrent fusions in B-cell acute lymphoblastic leukemia patients.	(114)
Breast cancer	Mexico, 2023	RNA-seq in 12 luminal breast cancer patients	Pseudoalignment to the reference transcriptome: Salmon* Differential expression: DESeq2*.	269 DEG in chemoresistant patients. *SLC12A1* expression and *GLUR4* protein levels could be linked to chemoresistance in luminal breast cancer.	(115)
Acute lymphoblastic leukemia	México, 2024	Affymetrix Human Transcriptome Array 2.0 was used to identify aberrant gene expressions in 43 bone marrow samples from adults with acute lymphoblastic leukemia.	Microarray gene expression data processing: Affymetrix Transcriptome Analysis Console*	A total of 871 DEG. Top upregulated genes were *DNTT, MYB, EBF1, SOX4*, and *ERG*, while *PTGS2, PPBP, ADGRE3, LUCAT1*, and *VCAN* were most downregulated. *ERG, CDK6*, and *SOX4* linked to relapse and mortality risk.	(116)
Breast cancer	Argentina, México, Brazil, Uruguay and Chile, 2024	340 patients with breast cancer HR+/HER2- on adjuvant therapy were analyzed using Agilent microarrays.	Agilent gene expression platform*	Transcriptomic risk classifiers were proved clinically valid and superior to clinical and immunohistochemistry methods in real-world node-negative HR+/HER2- tumors.	(112)

DEG, differentially expressed gene; *Bioinformatics algorithms/software.

## 3 Challenges and opportunities

Despite the transformative potential of cancer genomics in oncology practice and research, several challenges impede their widespread adoption in healthcare. Among these, limited human and computational resources hinder equitable access to genomic technologies, especially in low- and middle-income countries. Similarly, the lack of integration of genomic information into clinical practice and healthcare systems may slow its adoption. Given the growing importance of this field in medical practice, numerous opportunities have also emerged to tackle these challenges. Collaborative genomics initiatives and consortiums, for example, are powerful tools to optimize resource usage and reduce costs. Likewise, medical education and training are key to equipping healthcare professionals with the knowledge and skills needed for genomic-driven care. In this section, we will explore these topics in more depth and provide emphasis on the Latin American region.

### 3.1 Human and computational resources

The growing amount of cancer genomics data raises the need for specialized solutions able to provide efficient and reliable bioinformatics services to the clinical community. This increasing demand for bioinformatics solutions has long been recognized and plays an important role in current clinical settings (118). In order to provide these services, different resources should be considered, including computing platforms (infrastructure), algorithms and software, and human resources. Each of these levels has experienced significant progress and has accelerated the implementation of cancer genomics into the clinics.

At the computational infrastructure level, demands for handling, storing and analyzing massive amounts of genomic data can be solved on distributed high-performance computer (HPC) systems. HPC can be defined as a technology that uses supercomputers or computer networks, named clusters or grids, to process massive datasets and solve complex tasks at high speeds (119). Additionally, cloud storage and computing have emerged as powerful tools useful for data sharing and demand-driven computation, reducing costs, facilitating access and collaborations, and surpassing geographical and infrastructure barriers (120).

A second major area of concern includes software and algorithm development. These tools and methods are critical to optimizing data analysis and interpretation and constitute subjects of intense development and research. In variant calling, for example, new insights using artificial intelligence (AI)-based tools such as DeepVariant, developed by Google, and DRAGEN, developed by Illumina, have shown promising results (121, 122). Other efforts have been focused on developing software to improve interoperability and management of databases, this includes relational databases and standardization of genomic data for precision medicine. As an example, the Genomic Data Commons (GDC) platform developed by the National Cancer Institute (NCI) integrates renowned cancer genomics datasets, processing the data in reproducible bioinformatics pipelines and democratizing access to cancer genomics data (123). Other initiatives include the creation of APIs (Application Programming Interfaces) such as Beacon by GA4GH, developed to aid data sharing without compromising personal and sensitive information (124).

Last but not least, bioinformatics services require experts in the field not only to develop these tools but also to implement bioinformatics protocols and best practices, troubleshoot, and provide advice on the clinical interpretation of genomic data. Some large-scale projects, such as data integration between ICGC and TCGA in the Pancancer Analysis of Whole Genomes (PCAWG) study, for instance, required almost 1,300 researchers to complete bioinformatics tasks like consolidation of histopathological data, uniform data processing, variant calling, and quality control of somatic and germline variants from 2,600 cancer samples (125). Importantly, there is a critical need for bioinformatics expertise in healthcare and research, and although multiple solutions in education and training have been proposed to mitigate this shortage, several challenges remain to be addressed (126, 127).

Latin American countries have faced significant challenges in research and development, principally derived from insufficient and discontinuous funding that led to difficulties in maintaining and updating the necessary infrastructure, software and training for researchers to keep up with new technologies. These technological challenges arise from the need for standardization, the deployment of structured databases and the acquisition of advanced equipment, among others. Also, bioinformatics education and training are still considered an important challenge in Latin America and other low- to middle-income countries (LMICs) (128). In this regard, Argentina, Brazil, Chile, Colombia and Mexico have relatively advanced bioinformatics programs compared to other countries in Latin America and have shown successful results in integrating this field into basic and applied research (128–131). On the other hand, new computing paradigms related to decentralized and low-cost infrastructures, such as cloud computing, are alternatives to optimize available resources without compromising quality (120). Additionally, cooperative databases and open-source software and algorithms have brought opportunities to conduct high-quality research and offer cutting-edge bioinformatics services. Finally, international consortia and initiatives, explored below, have shown to be effective in optimizing the usage of these resources. Given the growing importance of cancer genomics bioinformatics in oncology, we highlight the importance of the allocation of resources and investment to improve the outcomes of patients with cancer.

### 3.2 National and international cancer genomics initiatives and consortiums

Advances in NGS and bioinformatics technologies have accelerated the generation of cancer genomics data, which in turn has been critical to understanding its molecular basis and the development of targeted therapies. Importantly, the number of patients/samples and the scale of projects aimed at studying such associations impose significant challenges in terms of large-scale patient recruitment, sample processing, data collection, and data analysis in a timely and resourceful manner. Cooperation arises as a powerful solution to tackle these problems under the figure of multicenter initiatives and consortia. In the scientific community, a consortium is defined as the association of a multidisciplinary group of scientists from diverse institutions and/or countries that collaborate on research efforts to achieve a common goal (132). In practical terms, these networks have contributed to the pooling of information, the development and validation of tools and the subsequent analyses in multicentric projects. Furthermore, consortia have served as a platform for training on genomic research and promotion of institutional infrastructure for data collection, analysis and sharing. One of the most significant examples of collaborative research worldwide was the Human Genome Project dedicated to establishing a standard sequence of the human genome by the International Human Genome Sequencing Consortium (133). Another illustrative example of recent large-scale cooperative efforts is the 100,000 Genomes Project, a British initiative aimed at sequencing the whole genome of patients from the United Kingdom National Health Service (NHS) affected by rare diseases and cancer (134). Importantly, these ambitious projects highlight the importance of interinstitutional and governmental cooperation to strengthen research capacities and optimize resources.

This kind of approach is particularly relevant in cancer genomics as it advances towards massive genomic and clinical data (GCD) analyses and fast-paced technological developments. Importantly, the integration of such data across different centers and institutions is necessary to achieve statistical power and obtain robust results in considerably shorter times, accelerating the implementation of precision oncology solutions (135). Global initiatives, such as the International Cancer Genome Consortium (ICGC) and Project GENIE (Genomics Evidence Neoplasia Information Exchange), created by the American Association for Cancer Research (AACR), are examples of relevant consortia in this area (136, 137). The ICGC was launched in 2008 as a large-scale collaborative effort to characterize genomic abnormalities among different cancer types using genomic, transcriptomic and epigenomic information (136). In 2019 the ICGC data portal contained data from 84 worldwide cancer projects, from 20,000 contributors and 77 million somatic mutations, including data from the Cancer Genome Atlas (TCGA) and the Sanger Cancer Genome projects. While the original web portal was available until June 2024, data remains available to researchers and the ICGC has advanced to a new phase, the ARGO (Accelerating Research in Genomic Oncology) project, an international initiative aimed at analyzing specimens from 100,000 cancer patients worldwide (136). Similarly, Project GENIE is a large international consortium aimed at catalyzing the sharing of GCD, enabling precision cancer medicine research (138). Launched in 2015, they have sequenced 214,487 samples from 184,988 patients and 18 contributing institutions until 2024. Remarkably, the utility of such initiatives is not limited to academic and research activities, but also to improving decision-making in oncology clinical practice.

In Latin American countries similar initiatives have been built over the years in an effort to ensure the representativity of such populations. Despite these advances, a clear underrepresentation of several ethnic groups, including Latin Americans, has been evidenced in medical and cancer genomics (139, 140). In addition to providing valuable information to fill this gap, these initiatives have successfully addressed multiple challenges, including limited funding, infrastructure and human resources (141). Most Latin American consortia are focused on studying specific cancer types and are funded and run by governmental and international organizations; examples of such efforts are presented in **Table 4**. Among some successful examples of such efforts, we can highlight the PRECAMA project, a large multicenter case-control study aimed at advancing the prevention and management of breast cancer in premenopausal Latin American women (142). This study is coordinated by the International Agency for Research on Cancer (IARC), enrolling patients from Chile, Colombia, Costa Rica, Mexico, and Brazil, and implementing a multidisciplinary approach that combines genetics, genomics, and metabolomics with lifestyle factors. Another example is the Latin American Consortium for Lung Cancer Research (CLICaP - “Consorcio Latinoamericano para la Investigación del Cáncer de Pulmón”), an initiative launched in 2011 by a network of Latin American oncologists to improve and promote clinical and translational research in lung cancer (143, 144). For 2021, this consortium included more than 75 researchers from most Latin American countries and has had a considerable impact on access to funding, coordination of multicenter research, number and quality of publications, and development of clinical guidelines adapted to a local context. Finally, another interesting example of collaboration in the region is the Brazilian Hereditary Tumors Study Group (145). Founded in 2003, the group initially published updates on hereditary cancer in Brazil with the mission of improving teaching and research into hereditary cancer and encouraging national and international collaboration. In 2007, numerous researchers and groups from other South American countries became interested in participating, widening its outreach and becoming the Study Group on Hereditary Tumors (GETH). Several publications and active participation and interaction of GETH members reflect the importance of local efforts to promote collaborations and partnerships (146, 147).

**Table 4:** Examples of initiatives and consortia in cancer genomics with participation of Latin American countries.

Name	Starting year	Included Latin American countries	Scope	Reference
International consortia led by IARC	1965	Brazil is included as a founding plus country, and they have a long-term collaboration in over 101 low- and middle-income countries (LMICs), several from LA.	To promote international collaboration in cancer research creating work teams of researchers from 8 different branches with focus on epidemiology, wet-lab and biostatistical skills to study cancer types of relevance for LMICs	www.iarc.who.int
Brazilian Study Group on Hereditary Tumors – (GBETH)	2003	Brazil, Argentina, Chile and Uruguay	To establish the South American Collaboration of Registries on Hereditary Cancer on a web platform, integrating clinical and molecular data	www.geth.org.br
Molecular Subtypes of Premenopausal Breast Cancer in Latin American Women (PRECAMA)	2007	México, Costa Rica, Colombia, Chile	Multicentric case-control study in Latin America that aims to advance the prevention and management of breast cancer in Latin America through a better understanding of the molecular, pathological, and risk factor patterns from the region.	https://precama.iarc.who.int
The Latin American Cooperative Oncology Group (LACOG)	2009	Located in Brazil with over 259 institutions from 16 Latin American countries	Created by oncologist physicians with the aim to develop observational studies and clinical trials on several types of tumors to improve expertise in the region.	https://lacogcancerresearch.org
Latin America Cancer Research Network (LARCN)	2009	Brazil, Mexico, Argentina. Chile, Uruguay	To support a clinical cancer research network in Latin America with collaborative agreements between Latin America and USA with an impact in colorectal, pediatric and breast cancer	(141)
Red Iberoamericana de bioinformática (SolBio)	2009	Argentina, Bolivia, Brazil, Chile, Colombia, Costa Rica, Cuba, Ecuador, El Salvador, Guatemala, Honduras, Mexico, Nicaragua, Panama, Paraguay, Peru, Puerto Rico, República Dominicana, Uruguay, Venezuela, plus Spain and Portugal.	Promotes bioinformatics and computational biology in the large region of Ibero América	https://wp.soibio.org
Consorcio Latinoamericano para la Investigación del Cáncer de Pulmón (CLICAP)	2011	México, Colombia, Brazil, Costa Rica, Argentina, Panamá, Peru, Venezuela, Uruguay, Nicaragua, Chile, Ecuador.	To positively impact the approach, care, and prognosis of lung cancer patients in Latin America through clinical research and continuing medical education.	https://clicap.org/
Variant Interpretation for Cancer Consortium (VICC) is a Driver Project of the Global Alliance for Genomics and Health.	2016	Brazil	Standardize and coordinate clinical-genomics curation efforts, to facilitate integration of the knowledge and evidence provided by institutions in academia, government, and industry alike.	https://cancervariants.org
Global Cancer Consortium	2020	Brazil	To foster global transdisciplinary collaborations in cancer education, research treatment and community outreach to overcome cancer burden.	https://glocacon.org
Fred Hutch/University of Washington/Seattle Children’s Cancer Consortium	2021	Brazil	To increase understanding, strengthen prevention, diagnostic capabilities, and develop effective therapies for cancer.	https://www.cancerconsortium.org
Latin America Genomics of Breast Cancer Consortium (LAGENO-BC)	2022	Brazil Uruguay Puerto Rico, Mexico, Argentina. Chile, Peru, Colombia, Nicaragua. They are a branch project from LARCN - See above	Aims to build a large research resource of genome wide genotype data including individuals from different countries and genetic ancestry proportions	https://www.lageno-bc.org
ISCB-Latin America SoIBio CCBCOL Conference on Bioinformatics	2024	Argentina, Bolivia, Brazil, Chile, Colombia, Costa Rica, Cuba, Ecuador, El Salvador, Guatemala, Honduras, Mexico, Nicaragua, Panama, Paraguay, Peru, Puerto Rico, República Dominicana, Uruguay, Venezuela, plus Spain and Portugal.	Aims to promote scientific and professional exchange in bioinformatics across Latin America.	https://www.iscb.org/latam2024/home

Regarding bioinformatics infrastructure initiatives, these have been essential to improve capacity building in other countries. For example, worldwide-known networks such as ELIXIR or BBMRI-ERIC offer several resources to support computational capabilities and maintain data repositories and biobank data (148, 149). They have also developed training resources for scientists/developers and created guidelines to allow interoperability between data and centers. In Latin America there are societies such as the CABANA Network, UNU BIOLAC and groups affiliated with SolBio (Iberoamerican Society for Bioinformatics) focused on accelerating the implementation of bioinformatics through training programs and research collaborations (150–152). However, in Latin America, data repositories are not interoperable between institutions, and there are no organized initiatives to share and store biological information. Latin America should walk toward an integrated and organized infrastructure to improve its role in global research (153).

### 3.3 Integration into the clinical practice and healthcare systems

There is a growing interest in genomic approaches in cancer research and clinical oncology. Over the past years, this interest has been translated into remarkable progress in cancer genomics worldwide, including Latin America, with Argentina, Brazil, Chile and Mexico leading the way in the region (154). The implementation of such approaches in clinical practice has been driven by numerous institutions, governmental actors, initiatives and consortia (155). While the importance of this integration is highlighted by the increasing usage of genomic biomarkers in oncology, several challenges regarding cost-benefit, clinical usefulness and precarious healthcare systems remain to be solved (42). Furthermore, these approaches have introduced new considerations such as increasing costs, the privacy of genomic data, and data sharing and harmonization, which will be increasingly important in the near future (156).

In some Latin American countries, cancer genomics profiling is currently part of the clinical practice to predict therapy response and identify relevant genetic variants (157). In Mexico, for example, the Genomic Diagnostic Laboratory, established at the National Institute of Genomic Medicine, offers comprehensive genetic testing services, analyzing genes linked to hereditary conditions and cancer predisposition syndromes (42). In Chile, a promising 25-gene NGS somatic panel called TumorSec™ has been recently developed and validated (84). This panel was designed to detect actionable mutations in tumors common in Latin America, including breast, colorectal, gastric, ovarian, pancreatic, and gallbladder cancers. This assay incorporates an automated bioinformatics analysis aiming to facilitate the implementation of precision medicine in Latin America by providing a cost-efficient alternative to multiple non-NGS assays and larger and more expensive NGS panels. In Colombia, cancer genomics research is currently centralized, with major medical centers such as the National Cancer Institute implementing NGS panels for the assessment of germline and somatic variants in cancer patients (158). Centers like these have assembled multidisciplinary teams of pathologists, geneticists and bioinformaticians to analyze genomic data, focusing on highly prevalent neoplasia such as breast, lung and colorectal cancer. The implementation of NGS technologies has unlocked new research opportunities, enabling a deeper understanding of the molecular epidemiology of these cancers and facilitating comparative studies with other populations.

Despite the advancements, the implementation of genomic cancer bioinformatics in Latin America is significantly constrained by multifaceted challenges, including pronounced geographical disparities that disadvantage rural areas, a concentration of healthcare professionals and technology in urban centers, and limited funding in the public sector. The high costs of targeted therapies and restricted availability of genomic platforms further deepen inequities in cancer treatment (159). Importantly, the region has been slower to adopt genomic technologies for routine use compared to other parts of the world. Significant challenges in funding and research infrastructure may explain this slow-paced adoption (154). In addition, navigating intricate and often inconsistent regulatory frameworks across countries can delay clinical trial approvals and implementation, hindering the region’s integration into global research efforts. The result is a complex landscape where precision oncology remains largely inaccessible to significant portions of the Latin American population, particularly in underserved and rural communities (160).

On the other hand, Latin America has not developed unified standards or guidelines to assess NGS technologies or bioinformatic procedures in healthcare and currently employs references from the US or Europe (e.g. AMP/ASCO/CAP guidelines, ESMO Scale for Clinical Actionability of molecular Targets - ESCAT). In general, most studies and laboratories have incorporated US guidelines, a trend also observed in clinical oncology in countries such as Brazil, Mexico, and Colombia (47, 161, 162). Interestingly, a growing number of studies aimed at strengthening research collaborations with European countries and organizations may change this trend (146, 163). With the growing importance of these technologies in healthcare in the region, it is worthwhile to develop and implement guidelines and protocols adapted to local settings and evidence-based, ideally based on transnational collaborations and with support of scientific societies and governmental agencies.

Other important barriers include insufficient funding for science and technology, expensive imported equipment, lack of local infrastructure, and a shortage of trained professionals. In fact, many institutions must send samples abroad for analysis, increasing costs and limiting flexibility. Some private laboratories, particularly in larger cities, offer advanced medical technologies as a service to hospitals that may not have these capabilities in-house. This arrangement allows smaller or less equipped hospitals to access cutting-edge diagnostic and treatment options without having to invest in expensive equipment themselves. Language barriers also exist, as many genomic analysis tools and educational programs are in English. To address these issues, experts recommend increasing regional collaboration, closer partnerships between hospitals and universities, improving government funding, developing local capacity, and creating resources in local languages (164). Similarly, several authors have highlighted the increasing importance of integrating genomic data into electronic health records, which is still incipient even in developed countries and requires an active effort to store, analyze, and share data relevant to oncologists and cancer researchers.

### 3.4 Medical education and training

NGS technologies and bioinformatics techniques have accelerated the adoption of cancer genomics in clinical oncology, allowing clinicians to deliver personalized treatments and improve diagnostics. However, one of the main challenges in this process is the knowledge translation of genomic information into clinical care by healthcare professionals. This issue is highly relevant as large amounts of new information and rapid technological advances are continuously transforming our understanding of cancer biology and its treatment. In order to face this challenge, numerous medical education training programs have highlighted the importance of the acquisition of abilities to obtain, understand, process, and use genomic information for cancer care-related decision-making, a concept termed cancer genomics literacy (43, 165). Numerous studies have explored this aspect among physicians and healthcare professionals. Ha et al., for example, analyzed 21 studies, 9 focused on cancer care, assessing three types of knowledge among the participants: awareness (general knowledge or perception), how-to (practical knowledge about the application) and principle knowledge (understanding of the theoretical principles) (43). Overall, the authors found that physicians’ knowledge about cancer genomics is limited. Interestingly, genomic literacy varied among specialty, location, years of practice, and type of genomic test, but even for oncologists, who felt more confident to communicate and interpret genomic results, an important percentage (~30%) did not feel confident with their knowledge about genomic tests. Another recent study also identified limited genomics training among physicians as an important barrier to the implementation of precision medicine in routine healthcare (166). Interestingly, this study found that 41% of physicians reported a lack of training to identify appropriate genetic tests and interpret their results. Another study in the UK, including approximately 10% of the country’s oncologists, found that 38.7% of them did not receive formal training in genomics, and 92.7% identified a need for additional genomics training (167). These studies highlight the urgent need to improve cancer genomics education among healthcare professionals.

Experts have proposed several solutions to enhance medical education and training in cancer genomics. The incorporation of genomic modules in undergraduate programs and specialized genomic training programs has shown successful results in bridging knowledge gaps in healthcare professionals and providing tools for continuous learning (168). In bioinformatics, an interesting approach focused on developing and delivering specialized workshops and courses for trainers (train-the-trainers) has been important in addressing the shortage of professionals in this area and could be applied to other emerging fields (127). Educational initiatives must also align with efforts to address infrastructure deficits. These efforts are particularly relevant in the Latin American context, where financial and healthcare resources are scarce, and the number of cancer genomics professionals is insufficient to meet the increasing demands. Furthermore, educational efforts must prioritize equity and accessibility, allowing professionals from different backgrounds to receive high-quality training and sustained mentorship (169). Finally, training programs should empower professionals to influence healthcare policies and promote regional collaborations (170). These strategies improve sustainability over time, stimulating the harmonization of standards, the implementation of good practices, and facilitating data sharing. Noteworthy, all these potential solutions require a substantial investment in education and training for clinicians at the undergraduate, graduate, and postgraduate levels, nevertheless, they constitute one of the backbones for future precision oncology.

Despite the limitations, several Latin American initiatives have been established to improve genomics education among healthcare professionals. The Latin American School of Human and Medical Genetics (ELAG) created by the Latin American Network of Human Genetics (RELAGH) in 2005, for example, has trained over 800 young researchers and professionals from 17 countries, emphasizing ethics and interdisciplinary collaboration in genomics (171). Similarly, organizations such as SOLFAGEN (Latin American Society of Pharmacogenomics and Personalized Medicine) and RELIVAF (Latin American Network for Implementation and Validation of Pharmacogenomic Clinical Guidelines) advocate for genomics and pharmacogenomics training opportunities to integrate genomic data into clinical practice (172, 173). Regarding policy advocacy, health organizations such as the Pan American Health Organization (PAHO) and the Americas Health Foundation (AHF) have emphasized the importance of creating genomic policies to improve access to precision oncology and shape public health strategies (160, 174). Additionally, leveraging existing groups and networks, such as RELAGH and national genetics and oncology societies, to strengthen educational programs and policies has shown to be useful in democratizing cancer genomics services and fostering collaborations (175). We strongly believe that education and knowledge are the basis for consolidating cancer genomics worldwide and should be prioritized in our region.

## 4 Future perspectives

**Figure 3:**
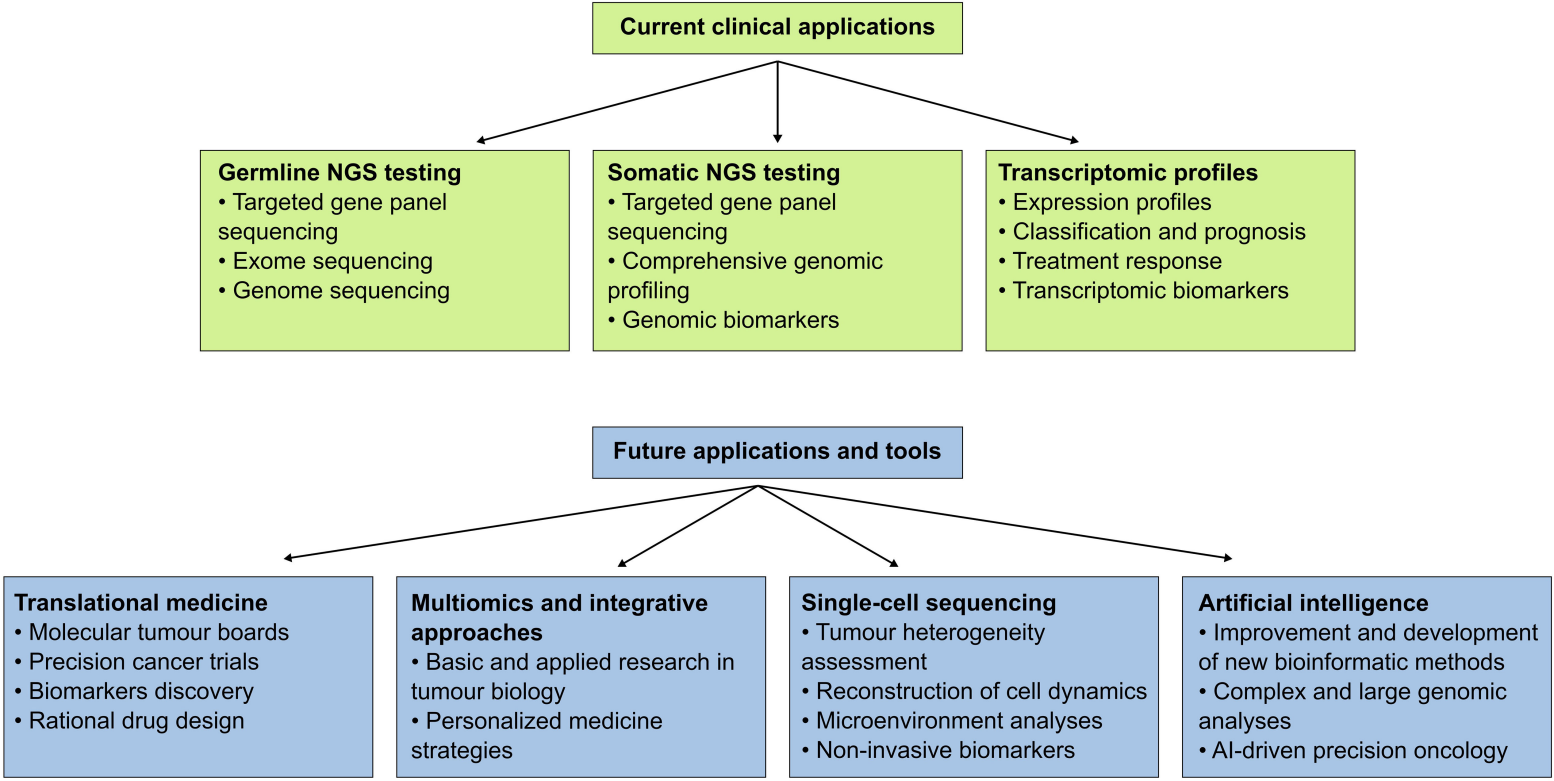
Current and future applications of NGS-based techniques in precision oncology and cancer research. Cancer genomics and bioinformatics are increasingly adopted in clinical settings; current applications include germline and somatic tests and transcriptomic profiles. Novel applications and tools in these areas are expected to improve cancer care and patient outcomes.

Cancer genomics is a promising field poised to transform cancer diagnosis, prognosis and treatment through the integration of cutting-edge technologies into clinical practice. In addition to current approaches, we anticipate that novel and exciting emerging areas will have increasing importance in clinical oncology (**Figure 3**). Translational medicine, for example, plays a pivotal role in bridging the gap between genomic discoveries and clinical applications, accelerating the development of personalized targeted therapies. Multiomics and integrative approaches are crucial for gaining a holistic understanding of tumor biology and are increasingly valuable in tumor classification and biomarker identification. Single-cell sequencing offers unprecedented insights into intratumoral heterogeneity and tumor evolution, enabling highly tailored treatment strategies. Additionally, artificial intelligence is revolutionizing data analysis, enhancing biomarker discovery and advancing precision oncology. This section will briefly introduce and discuss these topics.

### 4.1 Translational medicine in cancer

The ultimate goal of translational medicine in oncology is the development and application of new treatments, technologies and insights to improve cancer care and, ultimately, patient and population outcomes (176). Molecular data, including genomic information, has been critical in this process by enabling personalized treatments, improving early detection, and advancing targeted therapies in oncology. Furthermore, cancer genomics has also accelerated drug discovery and revealed mechanisms of resistance, improving treatment effectiveness (177). Among these transformative technologies, NGS has revolutionized cancer genomics by allowing comprehensive profiling of tumors at a scale and resolution previously unattainable. In addition to the current applications previously stated, novel NGS-based techniques promise improvements in cancer prevention and treatment. Numerous studies, for example, have identified the additive effect of multiple genetic variants associated with increased risk for tumors, underscoring the potential for polygenic risk scores to improve screening and prevention strategies (178). On the other hand, non-invasive tools such as NGS in liquid biopsies may facilitate early cancer detection and monitoring, while insights into tumor heterogeneity and evolution may guide adaptive therapy strategies (179). Similarly, the development and implementation of rapid and affordable sequencing methods and bioinformatics solutions have the potential to transform cancer research and clinical care (180).

The establishment of molecular tumor boards (MTB), which bring together multidisciplinary teams to discuss and interpret genomic data for individual patients, gathering laboratory experts and clinicians, has been a remarkable effort to accelerate the implementation of NGS in clinical practice (181). These boards play a crucial role in the implementation of precision medicine by ensuring that genomic insights inform diagnosis and treatment decisions. For instance, the Moffitt Cancer Center’s experience with its Molecular Tumor Board illustrates how such collaborative frameworks can effectively translate genomic findings into actionable clinical strategies (182). In line with these observations, other studies have shown the importance of these groups in selecting appropriate antitumor agents and guide therapeutic decisions, particularly for advanced and recurrent malignancies (183, 184). The integration of genomic data into clinical workflows also prioritizes the development of robust bioinformatics tools and databases that can effectively catalog and interpret the vast array of genomic alterations found in tumors. Resources such as GENIE, ARGO, JAX-CKB, and My Cancer Genome, among many others, provide clinicians with critical information on somatic mutations and their therapeutic implications, thereby facilitating the selection of appropriate clinical trials and targeted therapies (70, 138, 185, 186).

Despite their importance, MTB are not broadly implemented in oncology care across the Latin American region. A limited number of centers have begun to include MTB in the analysis of difficult cases. For example, an MTB in the Alexander Fleming Institute in Argentina was launched in December 2019 and by 2021 they have attended 32 challenging cases of different cancer types. Remarkably, for 87.5% cases a potentially actionable alteration was identified, from this group 47% received an approved or off-label treatment recommendation (187). Another example is the development of a virtual MTB strategy by the Foundation for Clinical and Applied Cancer Research (FICMAC) in Bogota, Colombia. This effort gave oncologists from different locations in Colombia the opportunity to submit clinical and laboratory records to a group of physicians including clinical oncologist, biologist, geneticist, pathologist and clinical study coordinators. Of the 146 patients included between 2020 – 2021, 53.1% received treatment recommendations based on genomic profile analyses (188). Even though MTB recommendations are not mandatory or broadly implemented, these reports are examples of the clinical potential of multidisciplinary expert panels to improve cancer care through precision oncology and cancer genomics.

Several translational cancer studies have been conducted in the Latin American region, with Argentina, Brazil, and Mexico emerging as leading contributors (189). A study conducted in Brazil, for example, successfully established a novel cervical cancer cell line derived from Brazilian individuals (190). Researchers performed whole-exome sequencing (WES) on these cell lines and applied advanced bioinformatics tools for comprehensive analysis. Interestingly, the authors identified potential new targetable biomarkers specific to the Brazilian population. Another multinational study among different Latin American countries analyzed the prognostic value of transcriptomic analyses in a large cohort of patients with locally advanced breast cancer, finding specific expression patterns and providing novel insights into new therapeutic approaches and precision oncology (110). Several other studies focused on preclinical and clinical models, population differences and international partnerships are illustrative of the potential of this approach (189). Given the complexity and costs associated with these studies, it is important that governments and private actors prioritize in a steady and active manner such efforts to optimize the potential clinical impact.

### 4.2 Multiomics and integrative approaches

In recent years, multiple technological advancements have shifted the paradigm of cancer research towards multi-omics analyses (191). These technologies enable a comprehensive and unbiased integration of multiple high-dimensional datasets, including genomic, epigenomic, transcriptomic, proteomic, and metabolomic data, among others. This comprehensive and integrated approach can characterize the multilayer intersections between different data types, creating an extensive understanding of biological profiles from cancerous tissue or individual cells of tumors and patients. In cancer research and clinical settings, having orthogonal data streams provides a more robust and accurate picture of biological systems, which have been particularly useful in tumor classification and the development and assessment of novel predictive and therapeutic models (192, 193). Furthermore, conclusions obtained from multi-omics studies have provided novel and valuable insights into tumor pathophysiology and treatment resistance (191, 194). This kind of integrated information bridges the gap between genotype and phenotype, revealing how genetic and molecular perturbations translate into observable traits and clinical outcomes.

Despite the significant advances, there are multiple challenges and limitations to be addressed in this field. Data integration and harmonization, meaning combining different datasets to maximize their compatibility and comparability, remains a critical aspect of complex data analysis (195). This issue becomes critical in cancer research, where different formats, dynamic ranges and analytical or experimental errors may vary considerably between patients and omic data. In order to tackle this problem, mathematical, statistical and computational methods, such as machine learning and deep learning, have been implemented to improve the analysis of large volumes of high-dimensional datasets (194, 196). Most of these techniques are based on statistical modeling, classification and feature selection methods. Some of the most successful algorithms in overcoming the difficulties mentioned above use a method termed robust network-based penalized estimation, examples include ENET (Elastic net) and LASSO (Least Absolute Shrinkage and Selection Operator), which have been useful in identifying gene expression regulators, biomarkers and relationships between functional levels (197). Importantly, it should be highlighted that the performance of each algorithm or model depends on the biological characteristics of the samples and the specific aims of the study.

By integrating multiple molecular datasets, multiomic approaches have shown substantial promise in cancer subtyping, enabling the identification of tumor subgroups with unique biological features previously not identified (191). In these approaches, data clustering has proven particularly valuable in tumors such as lung, breast, and gastric cancer. For example, recent studies have revealed the important role of the KEAP1/NFE2L2 axis in lung cancer, dysregulation of cellular signaling pathways in gastric cancer and metabolic shifts in breast cancer subtypes (198–200). Other multiomic studies have contributed to the identification of prognostic and predictive biomarkers, such as MMP11 (Matrix metalloproteinase 11) and APOBEC, and real-time monitoring of treatment responses through liquid biopsies in patients with lung cancer (191, 201). Furthermore, the identification of dysregulated pathways, such as HER2 signaling in breast cancer and MAPK in gastric cancer, has facilitated the development of targeted therapies (202, 203). In addition to deepening our understanding of cancer initiation and development, this integrative characterization of tumors accelerates the development of innovative therapeutic interventions tailored to the complexity of individual cancers.

Extending these contributions, multi-omics approaches are now paving the way for the development of novel therapies and personalized cancer treatments. Emerging technologies like spatial multi-omics and single-cell multi-omics may strengthen these efforts by analyzing tumor heterogeneity and tumor microenvironments, identifying spatially regulated biomarkers and refining drug delivery strategies (204, 205). Furthermore, other integrative frameworks such as pharmacogenomics and epitranscriptomics are advancing personalized treatments by tailoring therapies to individual genetic and molecular profiles, predicting responses and mitigating resistance (194). As multi-omics continues evolving and improving, its capacity to unravel the intricate biological underpinnings of cancer remains promising in transforming cancer drug discovery and precision oncology, ensuring that treatments are optimized for each patient’s unique molecular profile. Remarkably, there is a considerable gap between basic research and clinical practice and most multiomic approaches are limited to research settings worldwide. Multiomic research is still incipient in most Latin American countries, nevertheless, given its increasing importance in oncology, significant growth is expected in the near future. Overcoming the challenges associated with its translation and implementation into clinical practice requires coordinated efforts to enhance education, standardize practices, and improve accessibility to multi-omics technologies, ensuring that all patients benefit from the potential of these innovative approaches.

### 4.3 Single-cell sequencing

Advances in single-cell sequencing (sc-seq) technologies have enabled cancer researchers to uncover cellular heterogeneity, and tumor microenvironment and dynamics in an unprecedented manner (206). In addition to characterizing the molecular state of each cell within a tumor, these techniques allow the analysis of large cell populations, making them powerful tools to identify rare cell types and dissect the molecular features of cancerous and adjacent noncancerous cells. Interesting initiatives such as the Human Tumor Atlas Network (HTAN) and the Human Cell Atlas, for instance, have aimed to use these methods to better characterize the molecular features of human cancers (207, 208). Also, recently, a growing number of studies have shown the utility of these techniques in translational oncology. Pellechia et al., for example, demonstrated the feasibility of anticancer drug response prediction at the single-cell level using computational and *in vitro* approaches (209). Other studies have been focused on the identification of cancer biomarkers related to patient outcomes and response to immunotherapy using sc-seq data (210, 211). Altogether, these findings suggest that sc-seq could be a promising approach in future precision oncology.

Several techniques currently enable the isolation and sequencing of single cells or nuclei (212). In addition to fresh tissues, recent protocols allow the processing of frozen and FFPE samples (213). Also, novel methods such as single-cell and spatial multiomics are increasingly available and are transforming the study of cancer biology (214). Similar to other high-throughput technologies, data analysis of sc-seq experiments is challenging and remains an important bottleneck that has motivated the development of novel computational methods (215). Standardized bioinformatics pipelines involve preprocessing of reads, quality control, gene count matrix generation and data normalization to obtain datasets compatible with downstream analyses such as clustering, cell annotation and cell-cell communication inference (216). Importantly, data collection and processing remain demanding steps that hinder a wider implementation of these methods in research and clinical settings.

In Latin America, these technologies are still incipient, and several additional barriers have limited their adoption, including poor distribution channels for equipment and reagents, lack of specialized expertise and limited number of core research facilities (217). Remarkably, several international initiatives led by research institutions such as Wellcome Connecting Science and the Human Cell Atlas have conducted hands-on laboratory and bioinformatics training courses and workshops in Latin American countries in an effort to support global scientific equity (218, 219). Likewise, NGS implementation in routine clinical settings in Latin America, we anticipate that sc-seq will be increasingly relevant in the study of patients with cancer.

### 4.4 Artificial intelligence

In recent years, Artificial Intelligence (AI) has become an emergent technology able to significantly disrupt many fields of research and medical practice. The main goal of AI is the development of computer systems capable of performing tasks that typically require human intelligence, such as learning, reasoning, problem-solving, and language understanding (220). In cancer genomics, the integration of AI tools is proving highly useful in managing and interpreting the vast amounts of data generated by NGS platforms and related technologies. One relevant example is the data analysis in big data cancer projects such as the TCGA (221). Since its creation, the TCGA has produced approximately 2.5 petabytes of data, enabling researchers to analyze and uncover novel patterns in disease behavior that could significantly influence cancer outcomes. Recently, numerous studies have shown the potential of AI tools in analyzing such massive amounts of information, illustrating the potential that these techniques offer (222, 223).

Given the versatility of AI-based techniques, these have been used in basic and translational cancer genomics research (221, 224). Complex and challenging tasks such as the identification of mutational signatures in cancer genomics data, integrative multiomic analyses, reconstruction of intratumoral heterogeneity using bulk sequencing, and single-cell genomic analyses are examples of successful applications of these techniques (225–228). Furthermore, common and critical bioinformatics tasks have also adopted AI techniques showing promising results. Different AI-based approaches, for example, have been used to improve variant calling in tumor samples (229, 230). On the other hand, numerous state-of-the-art bioinformatics tools used to predict variant pathogenicity, such as REVEL, spliceAI and AlphaMissense, employ AI methods such as random forest and deep neural networks (231–233). One of the main advantages of these approaches is the capacity to incorporate datasets with a large number of features or covariates to improve specific tasks such as classification, regression or clustering. Other tools have even moved further, allowing variant interpretation and prioritization, for example, VarChat and CancerVar (234, 235). It should be noted that although these tools are promising and have enormous potential, they have several limitations and therefore must be supervised by healthcare and bioinformatics professionals.

In Latin America, several research groups have begun utilizing AI in cancer genomics. The increasing accessibility of NGS platforms has facilitated the generation of tumor-related genomic data, encouraging the deployment of AI models. Recent studies by López-Cortés and other researchers from Ecuador and Spain, for example, have used numerous machine learning methods to identify cancer-driving proteins and targeted drugs using multi-omics data (236, 237). In Colombia, the national project GLORIA aims to integrate histology-genomic analysis using deep-learning models with Latin American population data (238). A team from Brazil and the Dominican Republic compared the performance of different deep learning autoencoders for cancer subtype detection using multiomics data from TCGA datasets, demonstrating their potential for predicting patient subgroups and survival profiles, and identifying differentially expressed genes (239). In Mexico, a team used single-cell RNA sequencing data to train deep neural networks for detecting myeloid malignancies in Fanconi anemia patients (240). Their models identified malignant cells with high accuracy, revealing their origin in specific blood cell precursors and signs of immune evasion. While these studies are still emerging, they highlight a growing interest in leveraging AI to address region-specific challenges in cancer genomics (241). We strongly believe that beyond the limitations of implementing AI and other cutting-edge technologies in our region, they constitute powerful tools in cancer genomics and precision oncology.

## 5 Conclusions

Recent years have seen novel and important insights into cancer genomics and precision oncology. This progress is largely due to advances in NGS and bioinformatics techniques and has profoundly transformed how we care for and treat patients with cancer. Currently, a number of NGS-based methods are available in the clinic. These methods include germline and somatic testing, as well as transcriptomic studies, and allow the identification of diagnostic, prognostic, and therapeutic cancer biomarkers. Despite the importance of these studies in regional settings, Latin America remains lagging in the implementation of these technologies compared to countries with advanced healthcare systems. In addition, bioinformatics analyses remain critical yet challenging. In Latin American groups several groups have conducted NGS-based studies showing the feasibility and constraints of these methods. We identified challenges that limit its wider adoption, including limited technological and human resources, difficulties in the integration of genomic information into clinical practice and healthcare systems, and insufficient education and training in cancer genomics. We highlight the importance of national and international collaborations as well as the consolidation of multidisciplinary groups, such as molecular tumor boards, to accelerate the implementation of these techniques and translation of cancer basic research into clinical practice. While these challenges are not limited to the Latin American region, several local factors exacerbate them and we noticed considerable differences in the adoption of NGS techniques among different countries. Finally, given the rapid technological advances in these areas, we highlight the importance of emerging technologies such as the growing implications of transitional medicine in cancer, multiomic approaches, sc-seq and AI-driven data analysis. Altogether, these current methods and future directions have tremendous potential to accelerate the implementation of precision oncology and ultimately improve the outcome and quality of life of patients with cancer in the Latin American region and worldwide.
